# Pattern of failure and optimal treatment strategy for primary gastric diffuse large B-cell lymphoma treated with R-CHOP chemotherapy

**DOI:** 10.1371/journal.pone.0238807

**Published:** 2020-09-22

**Authors:** Hye Jin Kang, Han Hee Lee, Seung-Eun Jung, Kyung-Sin Park, Joo-Hyun O, Young-Woo Jeon, Byung-Ock Choi, Seok-Goo Cho

**Affiliations:** 1 Department of Radiation Oncology, Catholic University Lymphoma Group, Seoul St. Mary's Hospital, College of Medicine, The Catholic University of Korea, Seoul, Republic of Korea; 2 Department of Gastroenterology, Catholic University Lymphoma Group, Seoul St. Mary's Hospital, College of Medicine, The Catholic University of Korea, Seoul, Republic of Korea; 3 Department of Radiology, Catholic University Lymphoma Group, Eunpyeong St. Mary's Hospital, College of Medicine, The Catholic University of Korea, Seoul, Republic of Korea; 4 Department of Pathology, Catholic University Lymphoma Group, Seoul St. Mary's Hospital, College of Medicine, The Catholic University of Korea, Seoul, Republic of Korea; 5 Department of Nuclear Medicine, Catholic University Lymphoma Group, Seoul St. Mary's Hospital, College of Medicine, The Catholic University of Korea, Seoul, Republic of Korea; 6 Department of Hematology, Catholic University Lymphoma Group, Yeouido St. Mary's Hospital, College of Medicine, The Catholic University of Korea, Seoul, Republic of Korea; 7 Department of Hematology, Catholic University Lymphoma Group, Seoul St. Mary's Hospital, College of Medicine, The Catholic University of Korea, Seoul, Republic of Korea; European Institute of Oncology, ITALY

## Abstract

**Purpose:**

The optimal treatment for primary gastric diffuse large B-cell lymphoma (PG-DLBCL) is still unknown. We evaluated unfavorable prognostic factors and pattern of failure in PG-DLBCL to determine the optimal treatment strategy.

**Methods:**

Between April 2001 and November 2018, 120 patients with complete remission following rituximab plus cyclophosphamide, doxorubicin, vincristine, and prednisolone (R-CHOP) chemotherapy were retrospectively reviewed. According to the Lugano staging system, 80 patients (66.7%) had localized disease and 40 patients (33.3%) had advanced disease. A total of 93 (77.5%) patients had single gastric lesion and 27 (22.5%) patients had multiple gastric lesions. Ninety patients (75%) were treated with R-CHOP chemotherapy alone and 30 patients (25%) received R-CHOP chemotherapy with additional local treatment for gastric lesions.

**Results:**

The 5-year locoregional failure-free survival (LRFS), progression-free survival (PFS), and overall survival (OS) rates in patients treated with R-CHOP chemotherapy with local treatment were 100%, 100%, and 100%, respectively, whereas the LRFS, PFS, and OS rates in patients treated with R-CHOP chemotherapy alone were 86.3%, 78.2%, and 87.4%, respectively (*p* = 0.031, *p* = 0.095, and *p* = 0.025, respectively). During the follow-up period, 17 patients (14.2%) had disease recurrence. Only 3 of the 17 patients had relapse in a completely new site without relapse in the initial involved site. All, except 2, cases of local recurrence included gastric failure. In the multivariate analysis, performance status and number of gastric lesions were independent prognostic factors for treatment outcome.

**Conclusions:**

Patients with complete remission following R-CHOP chemotherapy showed a good prognosis. The main pattern of failure in patients with PG-DLBCL was local recurrence, especially in the stomach. Patients who received local treatment for gastric lesions showed improved gastric control. Therefore, in patients with unfavorable prognostic factors, we recommend R-CHOP chemotherapy with additional local treatment for gastric lesions.

## Introduction

The stomach is the most commonly involved extranodal site in non-Hodgkin lymphoma, and diffuse large B-cell lymphoma (DLBCL) is the most common histologic type (40%–70%) of gastric lymphoma [1, 2]. The common symptoms of gastric lymphoma at presentation are usually nonspecific symptoms similar to those of gastritis, including epigastric pain, nausea, vomiting, abdominal fullness, and indigestion [3].

Treatments for primary gastric DLBCL (PG-DLBCL) are varied and can include surgical resection, systemic chemotherapy, and radiotherapy (RT) [4]. The optimal treatment for PG-DLBCL still remains unclear. Rituximab, a chimeric anti-CD20 monoclonal antibody, has been commonly used as a treatment strategy for DLBCL. Numerous clinical studies have demonstrated that adding rituximab to cyclophosphamide, doxorubicin, vincristine, and prednisolone (CHOP) chemotherapy improves progression free-survival (PFS) and overall survival (OS). Therefore, this approach has become the gold standard treatment for general DLBCL [5–8]. The principles of treatment of PG-DLBCL follow those of general DLBCL [9]. PG-DLBCL has a predominantly localized stage and shows a good prognosis with appropriate treatment [10]. Rituximab plus CHOP (R-CHOP) chemotherapy results in a 5-year OS of 90% in patients with PG-DLBCL [4, 11].

However, some patients still have an unfavorable prognosis. Prognostic factors associated with treatment outcome are still controversial. Therefore, it is necessary to predict the subgroups with a high risk of relapse and provide additional treatment for patients receiving R-CHOP chemotherapy. In an attempt to clarify the issue and determine the optimal treatment strategy, we conducted a retrospective evaluation of the unfavorable prognostic factors and pattern of the initial disease failure in PG-DLBCL.

## Materials and methods

### Patients and ethics

After obtaining approval from the institutional review board (Seoul St. Mary’s Hospital, The Catholic University of Korea; reference number, KC19RESI0346), we retrospectively reviewed patient data. In this study all patients’ records were analyzed in a fully anonymized and de-identified manner and no researcher had access to patients’ personal information, thus written informed consent was waived. Between April 2001 and November 2018, a total of 152 patients had histologically confirmed DLBCL and were diagnosed with primary gastric lymphoma according to the criteria defined by D’Amore et al. [12]. In the case of DLBCL with systemic involvement, PG-DLBCL was considered when there was a main bulky mass in the stomach. Patients with refractory disease who did not achieve complete remission (CR) following R-CHOP chemotherapy, were not treated with the R-CHOP regimen, and had incomplete treatment records were excluded. A total of 120 patients with PG-DLBCL were included in the study. All patients underwent staging investigations, including physical examination; laboratory data analysis; contrast-enhanced computed tomography (CT) of the neck, chest, and abdomen; 18F-fluorodeoxyglucose (FDG) positron emission tomography (PET)/CT; bone marrow aspiration and biopsy; and esophagogastroduodenoscopy (EGD) with biopsy. According to the Lugano staging system for gastrointestinal lymphoma, stages I and II_1_ were classified as localized disease, whereas stages II_2_, IIE, and IV were classified as advanced disease [13]. Multiple gastric lesions were defined as the presence of ≥2 separated lesions in the stomach.

### Treatments

All patients received 6–8 cycles of R-CHOP chemotherapy. The total number of R-CHOP cycles was determined by the interim treatment response after 3 cycles of R-CHOP chemotherapy. Patients who achieved CR in the interim response evaluation received 6 cycles of R-CHOP chemotherapy, and those who achieved partial response or in whom the disease was stable received up to 8 cycles.

Furthermore, 90 patients (75%) were treated with R-CHOP chemotherapy alone, while the remaining 30 patients (25%) received R-CHOP chemotherapy with local treatment, such as surgical resection and RT for gastric lesions. Twelve patients (10%) underwent total or subtotal gastrectomy before or during R-CHOP chemotherapy for alleviation of symptoms, e.g., perforation, uncontrolled bleeding, and obstruction. All the 12 patients with surgical resection received 6 cycles of R-CHOP chemotherapy and did not receive RT to prevent duplication of local treatment. Another group of 18 patients (15%) received consolidative RT on the stomach after achieving CR to R-CHOP chemotherapy as part of upfront treatment. RT was started within 8 weeks after completion of R-CHOP chemotherapy. The median RT dose was 30.6 Gy (range, 30.6–42 Gy) at 1.8–2 Gy per fraction. The clinical target volume (CTV) is the whole stomach with or without involved perigastric lymph node. The planning target volume was created by adding 1.5–2 cm margin to CTV. With the development of RT technique in our institution, patients were treated with conventional external beam, 3-dimensional conformal, or intensity-modulated RT. The use of consolidative RT was independently decided by patients’ attending physicians and retrospectively collected by researchers.

### Response to treatment, follow-up and statistical analysis

Tumor response was evaluated by EGD, contrast-enhanced CT, and PET/CT every 3 cycles and 1 month after completion of R-CHOP chemotherapy. CR was defined as complete resolution of all lesions on CT or FDG avidity score of 1–3 on PET/CT according to the Deauville criteria (5-point scale) [14]. All patients underwent endoscopy with biopsies of the stomach to confirm CR. Patients were followed at 1 month after completion of treatment, every 3 months for the first 2 years, and every 6 months thereafter. Physical examination, laboratory data analysis, and CT were performed at each visit. EGD was performed annually.

The primary study endpoint was locoregional failure-free survival (LRFS). The secondary endpoints were PFS and OS. LRFS was defined as the duration from the date of diagnosis to the date of disease relapse in the initial involved site, death from any cause, or last follow-up visit if a patient was free of disease. PFS was defined as the duration from the date of diagnosis to the date of disease relapse in any site, death from any cause, or last follow-up visit if a patient was free of disease. OS was defined as the time from diagnosis to death from any cause or last follow-up visit. The survival curves of LRFS, PFS, and OS were calculated using the Kaplan–Meier method. Univariate analyses were performed using log-rank test to assess the prognostic factor related to survival. Potential prognostic factors with *p*<0.1 in the univariate analyses were included in the multivariate analyses. Multivariate analyses were performed using the Cox proportional-hazards model. All test results were two-sided. A *P-*value of <0.05 was considered statistically significant. All statistical analyses were performed using the R (version 3.4.4).

## Results

### Patient characteristics and treatment outcome

The patient characteristics are summarized in Table 1. The median age was 59 years (range, 21–87 years). Women slightly outnumbered men (53.3% vs. 46.7%). The Lugano stage at diagnosis was as follows: 80 patients (66.7%) had localized disease, and 40 patients (33.3%) had advanced disease. A total of 93 (77.5%) patients had single gastric lesion, and 27 (22.5%) patients had multiple gastric lesions. Patients treated with R-CHOP chemotherapy with local treatment showed statistically significant higher percentages of younger age, GCB type, bulky gastric lesion, and lower IPI score than those treated with R-CHOP chemotherapy alone.

**Table 1:** Patient characteristics.

Characteristics	Total (n = 120)	Without local Tx. (n = 90)	With local Tx. (n = 30)	*p*-value
	n (%)	n (%)	n (%)	
Age (years)				*0*.*011*
<60	62 (51.7)	40 (44.4)	22 (73.3)	
≥60	58 (48.3)	50 (55.6)	8 (26.7)	
Sex				*1*.*000*
Male	56 (46.7)	42 (46.7)	14 (46.7)	
Female	64 (53.3)	48 (53.3)	16 (53.3)	
Lugano stage				*0*.*264*
I/II_1_	80 (66.7)	57 (63.3)	23 (76.7)	
II_2_/II_E_/IV	40 (33.3)	33 (36.7)	7 (23.3)	
ECOG				*0*.*057*
0	54 (45.0)	35 (38.9)	19 (63.3)	
1	49 (40.8)	40 (44.4)	9 (30.0)	
≥2	17 (14.2)	15 (16.7)	2 (6.7)	
B symptom				*0*.*777*
Present	20 (16.7)	16 (17.8)	4 (13.3)	
Absent	100 (83.3)	74 (82.2)	26 (86.7)	
Lactate dehydrogenase				*0*.*915*
Normal	71 (59.2)	54 (60.0)	17 (56.7)	
Elevated	49 (40.8)	36 (40.0)	13 (43.3)	
Pathologic type				*0*.*018*
GCB type	48 (40.0)	33 (36.7)	15 (50.0)	
Non-GCB type	50 (41.7)	45 (50.0)	5 (16.6)	
Missing	22 (18.3)	12 (13.3)	10 (33.4)	
EBV				*0*.*152*
Positive	21 (17.5)	61 (67.8)	23 (76.7)	
Negative	84 (70.0)	19 (21.1)	2 (6.7)	
Missing	15 (12.5)	10 (11.1)	5 (16.6)	
Extranodal involvement				
1 (stomach only)	93 (77.5)	66 (73.3)	27 (90.0)	
≥2	27 (22.5)	24 (16.7)	3 (10.0)	
Bulky disease status				*0*.*039*
Non bulky (<5 cm)	45 (37.5)	39 (43.3)	6 (20.0)	
Bulky (≥5 cm)	75 (62.5)	51 (56.7)	24 (80.0)	
No. of gastric lesion				*0*.*900*
Single	93 (77.5)	69 (76.7)	24 (80.0)	
Multiple	27 (22.5)	21 (23.3)	6 (20.0)	
IPI score				*0*.*041*
<3	89 (74.2)	62 (68.9)	27 (90.0)	
≥3	31 (25.8)	28 (31.1)	3 (10.0)	

Abbreviations: ECOG, Eastern Cooperative Oncology Group; GCB, germinal center B cell-like; EBV, Epstein–Barr virus; IPI, international prognostic index

**Fig 1:**
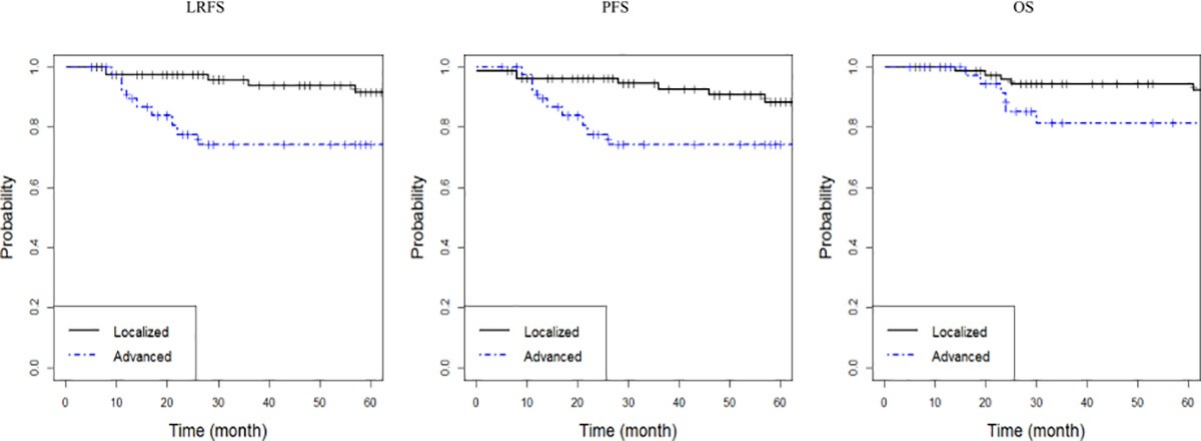
Kaplan–Meier curves of (A) locoregional failure-free survival (LRFS, *p =* 0.005), (B) progression-free survival (PFS, *p =* 0.009), and (C) overall survival (OS, *p =* 0.195) by Lugano stage.

The median follow-up duration was 49 months (range, 5–197 months). The estimated actuarial 5-year LRFS, PFS, and OS rates in all patients were 85.6% (95% confidence interval [CI], 78.7–93.7), 83.4% (95% CI, 76.1–91.5), and 90.3% (95% CI, 84.7–96.2), respectively. The 5-year LRFS, PFS, and OS rates in patients with localized disease were 91.5%, 88.3%, and 94.4%, respectively, whereas the LRFS, PFS, and OS rates in patients with advanced disease were 74.2%, 74.2%, and 81.3%, respectively (*p* = 0.005, *p* = 0.009, and *p* = 0.195, respectively; Fig 1).

**Fig 2:**
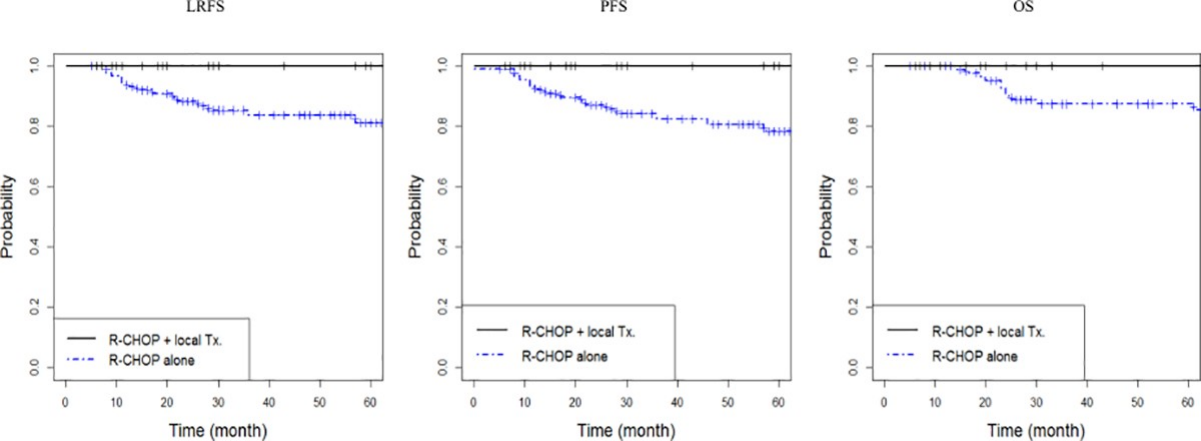
Kaplan–Meier curves of (A) locoregional failure-free survival (LRFS, *p* = 0.031), (B) progression-free survival (PFS, *p* = 0.095), and (C) overall survival (OS, *p* = 0.025) by treatment modality.

We also evaluated treatment outcomes according to treatment modalities and found that survival rates of patients who had received combined R-CHOP chemotherapy and local treatments were higher than those who received R-CHOP chemotherapy alone. The 5-year LRFS, PFS, and OS rates in patients treated with R-CHOP chemotherapy with local treatment were 100%, 100%, and 100%, respectively, whereas the LRFS, PFS, and OS rates in patients treated with R-CHOP chemotherapy alone were 86.3%, 78.2%, and 87.4%, respectively (*p* = 0.031, *p* = 0.095, and *p* = 0.025, respectively; Fig 2).

### Prognostic factors associated with treatment outcome

**Fig 3:**
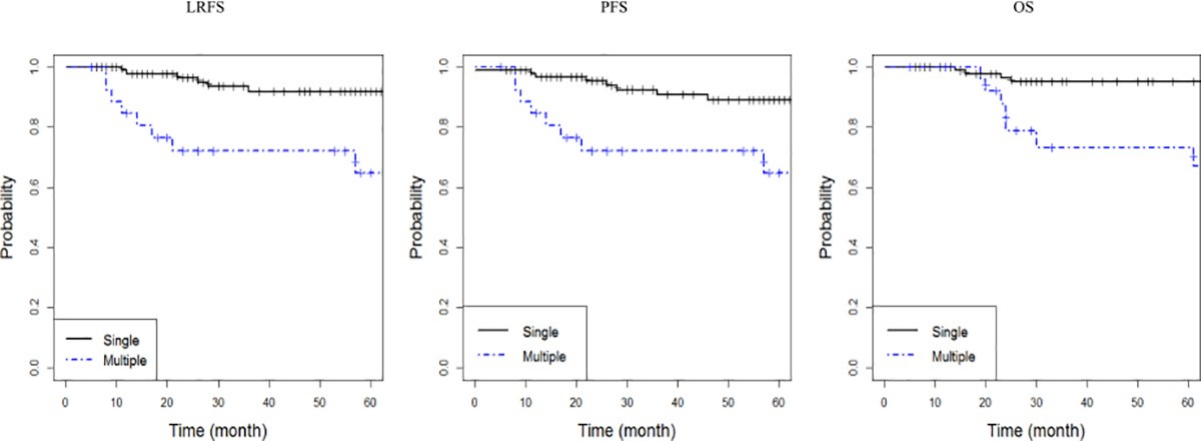
Kaplan–Meier curves of (A) locoregional failure-free survival (LRFS, *p*<0.001), (B) progression-free survival (PFS, *p* = 0.006), and (C) overall survival (OS, *p*<0.001) by number of gastric lesions.

In the univariate analysis, a lower LRFS rate was significantly strongly associated with old age (*p* = 0.042), advanced Lugano stage (*p* = 0.005), poor performance status (*p* = 0.040), non-germinal center B cell-like (GCB) type (*p* = 0.041), multiple gastric lesions (*p*<0.001), higher international prognostic index (IPI) score (*p* = 0.018), and R-CHOP alone without local treatment (*p* = 0.031). The lower PFS rate was associated with old age (*p* = 0.020), advanced Lugano stage (*p* = 0.009), poor performance status (*p* = 0.044), multiple gastric lesions (*p* = 0.006), and higher IPI score (*p* = 0.023). The treatment modality tended to be associated with the PFS (*p* = 0.095). The lower OS rate was associated with non-GCB type (*p* = 0.020), multiple gastric lesions (*p*<0.001), and R-CHOP chemotherapy alone without local treatment (*p* = 0.025). Detailed results of the univariate analysis on survival are shown in Table 2. In the multivariate analysis, the Eastern Cooperative Oncology Group (ECOG) performance status (hazard ratio [HR], 2.2; *p* = 0.038) and number of gastric lesions (HR, 6.59; *p*<0.001) were independent prognostic factors for LRFS. The number of gastric lesions was the only associated factor for PFS and OS (HR, 4.04, *p* = 0.005 for PFS; HR, 7.63, *p* = 0.001 for OS). The 5-year LRFS, PFS, and OS rates in patients with single gastric lesion were 91.8%, 88.9%, and 95.1%, respectively, whereas the LRFS, PFS, and OS rates in patients with multiple gastric lesions were 64.8%, 64.8%, and 73.2%, respectively (*p*<0.001, *p* = 0.006, and *p*<0.001, respectively; Fig 3).

**Table 2:** Univariate analysis for LRFS, PFS, and OS.

Variable	5-year LRFS (%)	95% CI	*p-*value	5-year PFS (%)	95% CI	*p-*value	5-year OS (%)	95% CI	*p-*value
Age (years)									
<60	91.7	84.0–100	*0*.*042*	89.3	80.7–98.9	*0*.*020*	92.0	84.6–99.9	*0*.*203*
≥60	79.0	67.8–92.0		77.3	65.9–90.5		85.5	76.1–96.1	
Sex									
Male	86.8	76.8–98.0	*0*.*810*	86.8	76.8–98.0	*0*.*636*	87.6	78.8–97.5	*0*.*108*
Female	84.8	75.6–95.2		80.8	70.7–92.5		92.7	86.0–99.9	
Lugano stage									
I/II_1_	91.5	84.4–99.2	*0*.*005*	88.3	80.3–97.1	*0*.*009*	94.4	89.1–99.9	*0*.*195*
II_2_/II_E_/IV	74.2	60.9–90.4		74.2	60.9–90.4		81.3	68.7–96.2	
ECOG									
0	95.3	89.2–100	*0*.*040*	92.4	84.3–100	*0*.*044*	97.7	93.4–100	*0*.*069*
1	81.0	69.6–94.2		65.8	44.5–97.4		86.1	76.3–97.2	
≥2	70.2	48.8–100		70.2	48.8–100		78.6	59.8–100	
B symptom									
Present	85.8	78.1–94.3	*0*.*650*	83.2	75.0–92.4	*0*.*506*	91.7	86.0–97.8	*0*.*512*
Absent	85.3	68.8–100		84.0	68.8–100		82.6	66.6–100	
Lactate dehydrogenase									
Normal	88.6	80.8–97.1	*0*.*352*	84.9	76.1–94.8	*0*.*838*	90.7	83.9–98.1	*0*.*822*
Elevated	81.2	69.2–95.3		81.2	69.2–95.3		89.7	80.6–99.8	
Pathologic type									
GCB type	90.8	81.4–100	*0*.*041*	87.7	76.1–99.8	*0*.*087*	97.4	92.6–100	*0*.*020*
Non-GCB type	75.4	62.2–91.3		73.5	60.2–89.6		82.9	72.1–95.4	
Extranodal involvement									
1 (stomach only)	87.9	80.5–95.9	*0*.*129*	85.2	77.2–94.0	*0*.*107*	90.3	84.1–96.9	*0*.*762*
≥2	77.6	61.8–97.4		77.6	61.8–97.4		90.4	78.6–100	
Bulky disease status									
Non bulky (<5 cm)	88.8	78.9–99.9	*0*.*389*	85.3	73.9–98.3	*0*.*410*	90.6	82.2–99.8	*0*.*557*
Bulky (≥5 cm)	83.8	74.8–93.9		82.4	73.3–92.8		90.1	82.8–98.0	
No. of gastric lesion									
Single	91.8	85.7–98.4	*<0*.*001*	88.9	81.9–96.6	*0*.*006*	95.1	90.6–99.9	*<0*.*001*
Multiple	64.8	47.1–89.4		64.8	47.1–89.4		73.2	56.8–94.5	
IPI score									
<3	89.4	82.0–97.5	*0*.*018*	86.5	78.3–95.5	*0*.*023*	93.5	88.2–99.2	*0*.*521*
≥3	74.1	59.1–92.8		74.1	59.1–92.8		79.7	65.3–97.3	
Treatment modality									
Without local Tx.	83.5	75.7–92.1	*0*.*031*	78.2	69.0–88.8	*0*.*095*	87.4	80.3–95.0	*0*.*025*
With local Tx.	100			100			100	100	

Abbreviations: LRFS, locoregional failure-free survival; PFS, progression-free survival; OS, overall survival; ECOG, Eastern Cooperative Oncology Group; GCB, germinal center B cell-like; IPI, international prognostic index; CI, confidence interval

### Patterns of failure and salvage treatment

During the follow-up period, 17 patients (14.2%) had disease relapse. The median time interval to relapse was 13 months (range, 4–80 months). A detailed pattern of the initial failure for individual cases is shown in Table 3. Seven patients had localized disease and 10 patients had advanced disease. Only 3 of the 17 patients had relapse in a completely new site without locoregional failure in the initial involved site. The sites of relapse were the lymph node (*n* = 2) and brain (*n* = 1). Furthermore, 14 of the 17 patients had the first relapse in the initial involved site, except in 2 patients; the site of relapse was the stomach with or without regional lymphatics. All disease relapses in patients with multiple gastric lesions occurred in the stomach.

**Table 3:** Pattern of failure for individual case.

No.	Age/sex	Stage	ECOG	IPI	Pathologic type	Gastric lesion	Extranodal involvement	Therapy	Time to failure (months)	Recurrence site
1	66/F	I	1	1	GCB	Single		R-CHOP 50%	24	Stomach
2	83/F	I	1	1	GCB	Single		R-CHOP 50%	31	Stomach
3	53/F	I	0	0	GCB	Single		R-CHOP 100%	42	Cervical LN*
4	63/F	I	1	1	Non-GCB	Single		R-CHOP 100%	9	Brain*
5	45/M	II_1_	2	2	Non-GCB	Multiple		R-CHOP 100%	53	Stomach
6	64/F	II_1_	1	1	Missing	Multiple		R-CHOP 100%	4	Stomach
7	37/M	II_1_	1	1	Non-GCB	Multiple		R-CHOP 100%	4	Stomach
8	65/F	IV	1	3	Non-GCB	Multiple		R-CHOP 100%	13	Stomach, cervical LN
9	63/M	IV	1	2	Non-GCB	Multiple		R-CHOP 100%	10	Stomach
10	72/M	IV	1	3	Non-GCB	Single		R-CHOP 100%	6	Stomach, pelvic LN
11	72/M	IV	1	4	Non-GCB	Single	Ileum	R-CHOP 100%	8	Stomach
12	62/F	IV	0	4	Non-GCB	Multiple	Liver, GB	R-CHOP 75%	16	Stomach
13	54/F	IV	0	2	GCB	Single	Spleen, lung	R-CHOP 100%	23	Spleen
14	64/F	IV	2	4	Non-GCB	Multiple	Colon	R-CHOP 50%	5	Stomach
15	69/F	IV	2	3	Non-GCB	Single		R-CHOP 50%	14	Left. iliac LN
16	52/M	IV	2	4	Non-GCB	Multiple	Spleen, mesentery	R-CHOP 100%	4	Stomach, mesentery
17	66/M	IV	1	3	GCB	Single	BM	R-CHOP 100%	80	Cervical LN*

Abbreviations: ECOG, Eastern Cooperative Oncology Group; IPI, international prognostic index; GCB, germinal center B cell-like; R-CHOP, rituximab, cyclophosphamide, doxorubicin, vincristine, and prednisone

*Isolated relapse in a completely new site

Furthermore, 15 of the 17 patients with relapse were treated with either salvage RT or second-line chemotherapy (i.e., dexamethasone, cytarabine, and cisplatin and ifosfamide, cisplatin, and etoposide). Only 5 patients achieved CR following salvage treatment, while the others had disease progression despite the intensive salvage treatment.

## Discussion

According to the treatment of general DLBCL, R-CHOP chemotherapy is the backbone treatment for PG-DLBCL. We performed R-CHOP chemotherapy as the first-line treatment in all patients with PG-DLBCL in the study. Although approximately one-third of the patients had stages II_2_ to IV, which was a relatively high proportion of patients with advanced disease, consistent with those in previous reports published in the rituximab era, the 5-year OS rate was 90.3% [4, 11]. We evaluated the prognostic factors and pattern of failure in patients with stages I to IV PG-DLBCL achieving CR following R-CHOP chemotherapy.

Age, Lugano stage, pathologic subtype, IPI score—well-known prognostic factors identified in several previous studies [15–17]—had statistically significant results in the univariate analysis but lost significance in the multivariate analysis. Additional local treatment to the stomach also showed a significant difference in treatment outcomes only in the univariate analysis. Rather than showing a truly low significance, this finding might reflect a lack of statistical power due to the small number of relapse cases. The multivariate analysis identified that the ECOG performance status and number of gastric lesions were the only independent prognostic factors. Poor ECOG performance status predicted significantly worse LRFS (HR, 2.15; *p* = 0.038) and marginally worse PFS (HR, 1.72; *p* = 0.098) and OS (HR, 1.99; *p* = 0.064). It was considered to provide insufficient dose of R-CHOP chemotherapy to control patients with poor performance status. Only half of patients with grade 2 ECOG performance status received the full dose of R-CHOP chemotherapy, while the other half received attenuated dose from 75% to 50% of the full dose. Similar to our results, Lin et al. also demonstrated that a better ECOG performance status was related to better PFS and OS by enabling more intensive chemotherapy regimen and adequate cycle of chemotherapy to be performed in patients with DLBCL [18]. Furthermore, in line with previously published results [19, 20], multiple gastric lesions were also identified for LRFS (HR, 6.59; *p*<0.001), PFS (HR, 4.04; *p* = 0.005), and OS (HR, 7.63; *p* = 0.001). For instance, Liu et al. reported that multiple gastric lesions were an independent adverse prognostic factor of OS (*p* = 0.011) [19]. Furthermore, Ishikawa et al. proposed a prognostic model of primary gastric DLBCL using the adverse prognostic factor, including multiple gastric lesions [20].

We found notable results in the site of relapse regardless of the pathologic subtype. About 80% of patients had failure at the initial involved sites even after achieving CR, 90% of them had gastric failure. Moreover, all disease relapses in patients with multiple gastric lesions occurred in the stomach (Table 3). Our findings are consistent with previously published data where the most common pattern of initial failure was local relapse following systemic therapy alone, compromising 44%–63% of all of disease failure cases [21–24].

A quarter of patients in this study underwent additional local treatment for gastric lesions. Patients who received local stomach treatment showed significantly improved gastric control compared with those who received R-CHOP chemotherapy alone at the 5-year follow-up (100% vs. 88.2%, *p* = 0.044). The benefits of local treatment on local control were also reported in a study by Li’s et al [9]. The authors achieved 100% local control in patients treated with consolidative RT at the 10-year follow-up (*p* = 0.028).

The pattern of failure and treatment outcome according to treatment modalities suggest a potential role for local treatment of the stomach. In the present study, surgical resection or consolidative RT was used as a local treatment for the stomach. Until the early 2000s, the surgical approach to gastric lesion was the standard treatment procedure for curative aim. However, postoperative complications, such as dumping syndrome, malabsorption syndrome, and impairment of quality of life, were constantly reported [25]. Moreover, systemic therapy with consolidative RT showed equivalent treatment outcomes but lower treatment-related morbidity compared to those of the surgical approach [10, 26]. No major acute and late RT-related toxicities, including gastric complication, liver dysfunction, and renal insufficiency, were found when consolidative dose was delivered to the stomach in a study conducted by Liu X et al. [15]. Surgical resection should be limited in treating complications and has been replaced by the conservative treatment approach. In our data, gastrectomy was performed in 12 patients only for palliative purposes before or during R-CHOP chemotherapy e.g., perforation, uncontrolled bleeding, and obstruction. Moreover, another 18 patients received consolidative RT on the stomach when they achieved CR after R-CHOP chemotherapy. The use of consolidative RT was independently decided by patients’ attending physicians. Although it was difficult to obtain detailed records of RT-related toxicity in our retrospective data, all patients completed the full course of RT, even those of advanced age or with poor ECOG performance status without interruptions due to RT-induced toxicities.

Additionally, only 5 of the 15 patients (33.3%) who received salvage treatment after relapse achieved CR, and the remaining 10 patients were unable to prevent disease progression despite the use of salvage treatment. Therefore, a more certain type of therapeutic strategy is required from the initial treatment of patients at a high risk of relapse.

Therefore, most patients with PG-DLBCL showed favorable treatment outcomes, and R-CHOP chemotherapy alone was sufficient. However, a more intensive treatment was required in subgroups with a high risk of relapse. Therefore, in patients with unfavorable prognostic factors, such as multiple gastric lesions, dose attenuation of R-CHOP chemotherapy for poor performance status, or old age, we recommend R-CHOP chemotherapy with additional local treatment to the stomach, such as consolidative RT even after achieving CR. Further prospective studies with a large sample size are needed to help illuminate the role of local treatment to the stomach in patients with PG-DLBCL.
